# OutbreakFinder: a visualization tool for rapid detection of bacterial strain clusters based on optimized multidimensional scaling

**DOI:** 10.7717/peerj.7600

**Published:** 2019-08-28

**Authors:** Ming-Hsin Tsai, Yen-Yi Liu, Chih-Chieh Chen

**Affiliations:** 1 Institute of Population Health Sciences, National Health Research Institutes, Miaoli, Taiwan; 2 Center for Research, Diagnostics and Vaccine Development, Centers for Disease Control, Ministry of Health and Welfare, Taichung, Taiwan; 3 Institute of Medical Science and Technology, National Sun Yat-sen University, Kaohsiung, Taiwan; 4 Rapid Screening Research Center for Toxicology and Biomedicine, National Sun Yat-sen University, Kaohsiung, Taiwan

**Keywords:** Multidimensional scaling, Microbiome clustering, Affinity propagation, Disease outbreak detection, MDS

## Abstract

With the evolution of next generation sequencing (NGS) technologies, whole-genome sequencing of bacterial isolates is increasingly employed to investigate epidemiology. Phylogenetic analysis is the common method for using NGS data, usually for comparing closeness between bacterial isolates to detect probable outbreaks. However, interpreting a phylogenetic tree is not easy without training in evolutionary biology. Therefore, developing an easy-to-use tool that can assist people who wish to use a phylogenetic tree to investigate epidemiological relatedness is crucial. In this paper, we present a tool called OutbreakFinder that can accept a distance matrix in csv format; alignment files from Lyve-SET, Parsnp, and ClustalOmega; and a tree file in Newick format as inputs to compute a cluster-labeled two-dimensional plot based on multidimensional-scaling dimension reduction coupled with affinity propagation clustering. OutbreakFinder can be downloaded for free at https://github.com/skypes/Newton-method-MDS.

## Introduction

Phylogenetic trees are widely used in public health to infer the molecular epidemiology of infectious diseases (Hall & Barlow, 2006; Pybus, Fraser & Rambaut, 2013). Many traditional methods such as the maximum likelihood, neighbor-joining, and Bayesian methods have been used to compute phylogenies for successful inference of epidemiological relationships (Den Bakker et al., 2014; Grad et al., 2012; Leekitcharoenphon et al., 2016; Tettelin et al., 2014). A phylogenetic-tree-like approach named “genetic relatedness tree,” constructed using pulsed-field gel electrophoresis (PFGE) and multilocus sequence typing (MLST) data, is widely used in molecular epidemiology (Hunter et al., 2005; Maiden et al., 1998). Deep learning has become more and more widely used to solve many of the most advanced issues in various fields. It can also be applied to bioinformatics to reduce the need for feature extraction and achieve high performance (Le & Nguyen, 2019). In epidemiological data analysis, the most crucial step in interpreting a phylogenetic tree is investigation of “clusters,” which are usually considered probable outbreaks (Ragonnet-Cronin et al., 2013; Vrbik et al., 2015). Evolutionary biologists can easily interpret phylogenetic trees; however, epidemiologists find it difficult to use phylogenetic information to assist their research on outbreak detection. Therefore, we introduced a dimension reduction approach named multidimensional scaling (MDS) to transform a phylogenetic tree into a two-dimensional plot and subsequently apply affinity propagation (AP) (Frey & Dueck, 2007) for clustering. This approach can facilitate rapid identification of relationships among compared bacterial isolates for investigation of clusters. In this paper, we present a tool called OutbreakFinder that can assist in outbreak detection; this tool can help users to plot an MDS plot with a cluster labeled from a distance matrix or alignment files from Lyve-SET (Katz et al., 2017), Parsnp (Treangen et al., 2014), or ClustalOmega (Sievers & Higgins, 2014).

## Materials and Methods

### Multidimensional scaling method

Multidimensional scaling is a widely used approach to projecting high-dimensional data onto a low-dimensional space, where the relationships among individual samples are preserved. This characteristic of MDS is often used to present high-dimensional data on a two-dimensional plane. A simple derivation of the MDS method is presented as follows:

Given *n* points in a *p*-dimensional space, the Euclidean distance between any two points, *x_r_* and *x_s_*, can be defined as $d_{rs}^2 = \mathop \sum \nolimits_{i\, = \,1}^p {\left( {{x_{ri}} - {x_{si}}} \right)^2}$. The MDS method aims to minimize the objective function as follows:

$${\rm{tress = }}\sqrt {{{\sum {^{{{\left( {{d_{rs}} - {{\hat d}_{rs}}} \right)}^2}}} } \over {\sum {^{^{{d_{rs}}}}} 2}}} ,$$

where ${\hat d_{rs}}$ is the predicted Euclidean distance. Let *B* = *XX^T^* be an *n* × *n* inner product matrix. In metric MDS, matrix *B* can be decomposed into *QMQ^T^*, where *Q* is an eigenvector and *M* is a diagonal matrix of eigenvalues. The two forms of matrix *B* are combined as follows:

$$QM{Q^T} = Q\sqrt M \sqrt M {Q^T} = {\left( {\sqrt M {Q^T}} \right)^T}\left( {\sqrt M {Q^T}} \right) = {X^T}X$$

Then, we obtain $X = \sqrt M {Q^T}$. Therefore, the aforementioned derivation demonstrates that the traditional MDS method is designed to find an approximate solution rather than the optimal solution. For detailed derivation of the traditional MDS method, please refer to Supplemental Appendix A.

### MDS by using Newton’s method

The 100 points in Fig. S1 are generated by the function genSimulationData() as shown in Supplemental Appendix B, every 10 points is a group, 0–9, 10–19, 20–29…, and so on. Figure S1A is an example of the distortion produced by the traditional MDS method, as shown in Supplemental Appendix C. Points 0–9 belong to the same group; however, points 9 and 3 evidently do not belong to the same group in traditional MDS (Fig. S1A). Therefore, we propose to perform MDS by using Newton’s method (NMDS) to compensate for the deficiencies of the traditional MDS method. The proposed method has two parts; one is NMDS and the other is determination of the largest error edge to be amended. The detailed steps of the algorithm are as follows:

1. ${\hat d_{jk}} = \sqrt {\mathop \sum \nolimits_{i = 1}^p {{\left( {{x_{ji}} - {x_{ki}}} \right)}^2}} $
2. ${r_{jk}} = {d_{jk}}/{\hat d_{jk}}$, where *d_jk_* is the observed distance
3. ${\hat x_{ji}} = \frac{1}{n}\mathop \sum \nolimits_{k = 1}^n \left( {{x_{ji}} - {x_{ki}}} \right)\left( {{r_{jk}} - 1} \right)$
4. Find the maximum error edge and force it to move to an ideal position (find the maximum ratio of ${\hat d_{jk}}/{d_{jk}}$ as the maximum error edge)

Here, ${\hat d_{jk}}$ represents the predicted distance between nodes *j* and *k*; *r_jk_* represents the ratio of the observed and predicted distance between nodes *j* and *k*, namely ${d_{jk}}/{\hat d_{jk}}$; ${\hat x_{ji}}$ represents the new value of dimension *i*; and *x_ji_* represents the current value of dimension *i*.

Steps 1–3 in NMDS minimize the objective function. Step 4 finds the maximum error edge and forces it to move to an ideal position. As shown in Fig. S1B, the proposed method can reduce grouping errors.

Multidimensional scaling by using Newton’s method is implemented in OutbreakFinder, which provides parameters through which a user can change the distance scale between data points, such as using a logarithmic scale or power 2 scale for distances. Users can specify the color of each data point or use the AP algorithm to cluster and specify the color of each group. In addition, OutbreakFinder generates a text file of data point coordinates and color, as well as an MDS plot graph. Users can reproduce the MDS plot figure by using other tools. OutbreakFinder can directly read Lyve-SET, Parsnp, and multiple sequence alignment files and plot MDS graphs. If a user does not use the aforementioned tools, he or she can generate a distance matrix to plot an MDS graph.

### Implementation

OutbreakFinder is written in Java and compiled into a standalone executable jar file that can be executed in Java Runtime Environment 1.8 or a later version. In addition, users can download the source code and compile it into a preferred version.

**Figure 1:**
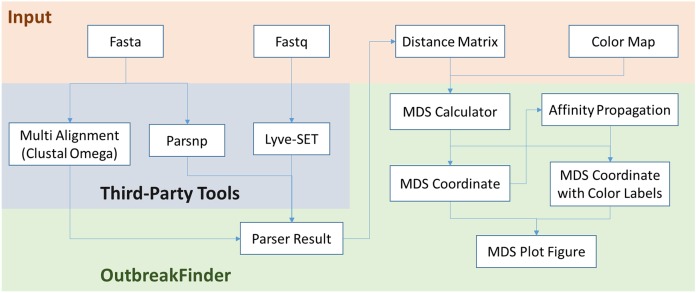
The schematic workflow of OutbreakFinder.

OutbreakFinder can parse the results of the Lyve-SET, Parsnp and Multi Alignment tools and generate MDS coordinates and graphs, as shown in Fig. 1. The user can define the color of each data point, and OutbreakFinder will export the corresponding image file according to the defined color map. Users can also use the AP module in OutbreakFinder for automatic clustering. The advantage of using an AP module for clustering is that there is no need to provide the number of groups, which makes cluster analysis easier.

### Example analysis

**Figure 2:**
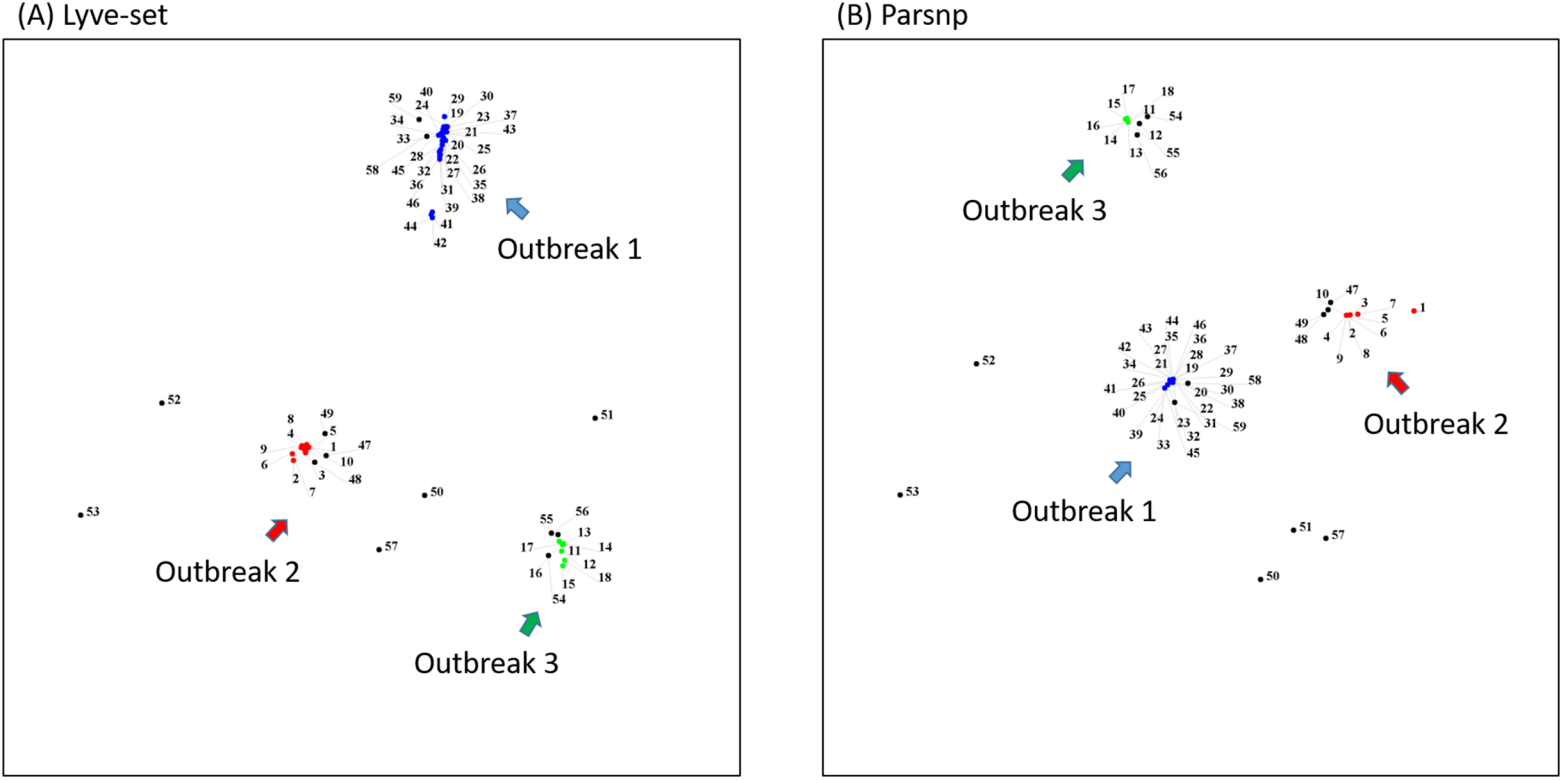
MDS plots of the 59 *Salmonella* Heidelberg isolates. The 59 *Salmonella* Heidelberg isolates from three outbreaks were identified as distinct clusters in the MDS plot by using Lyve-SET (A) and Parsnp (B). Outbreak 1, Outbreak 2, and Outbreak 3 are colored blue, green, and red, respectively.

**Figure 3:**
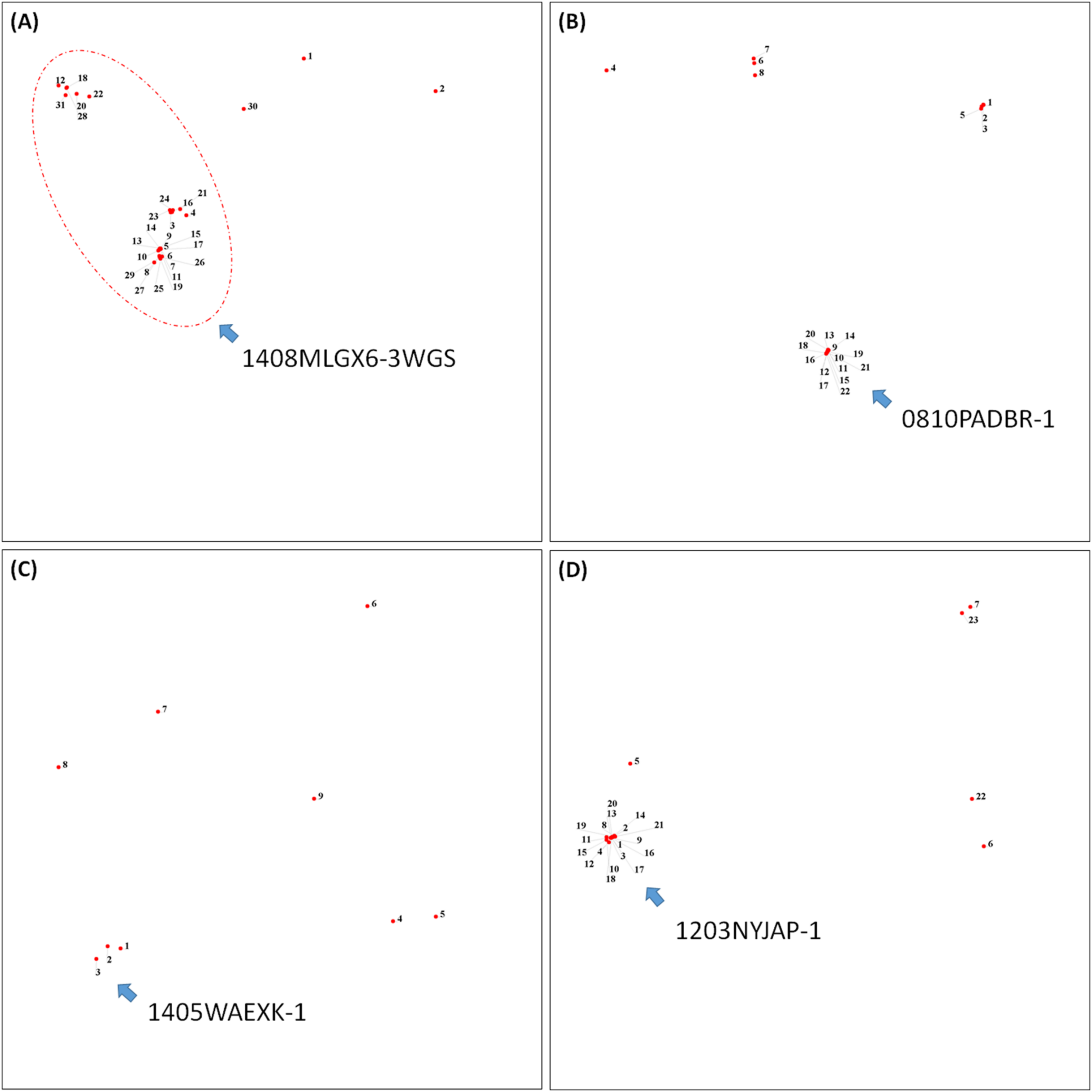
Results of the benchmark datasets. (A) Thirty-one *Listeria monocytogenes* isolates, (B) 22 *Campylobacter jejuni* isolates, (C) Nine *Escherichia coli* isolates, and (D) 23 *Salmonella enterica* isolates from outbreaks and outgroups were identified as distinct clusters in the MDS plot.

To demonstrate how to use OutbreakFinder, we employed 59 *Salmonella* Heidelberg isolate genomes from three outbreaks with the same PFGE type (Bekal et al., 2016). Whole-genome sequencing raw reads (Table S1) were downloaded from the National Center for Biotechnology Information’s Sequence Read Archive database (https://www.ncbi.nlm.nih.gov/sra). The downloaded sra files were converted into fastq files; then, Lyve-SET was used to extract SNPs from these fastq files and produce a distance matrix. OutbreakFinder uses the distance matrix to generate an MDS plot, as shown in Fig. 2A. Subsequently, we used the same dataset to test Parsnp. OutbreakFinder can directly parse the results of Parsnp output and generate an MDS plot, as shown in Fig. 2B. We use published benchmark datasets (Timme et al., 2017) to verify the availability of OutbreakFinder. This dataset contains four major foodborne bacterial pathogens, such as *Listeria monocytogenes*, *Campylobacter jejuni*, *Escherichia coli*, and *Salmonella enterica*. These pathogens are listed in Tables S2–S5. The procedure is the same as described above, Lyve-SET was used to extract SNPs and produce the distance matrixes, and then OutbreakFinder uses the distance matrixes to export the MDS plot. As shown in Fig. 3, outbreaks and outgroups can be clearly distinguished from the MDS plot. Although there are two outbreak clusters in Fig. 3A, the outbreak and outgroup can still be clearly distinguished.

### Performance

**Figure 4:**
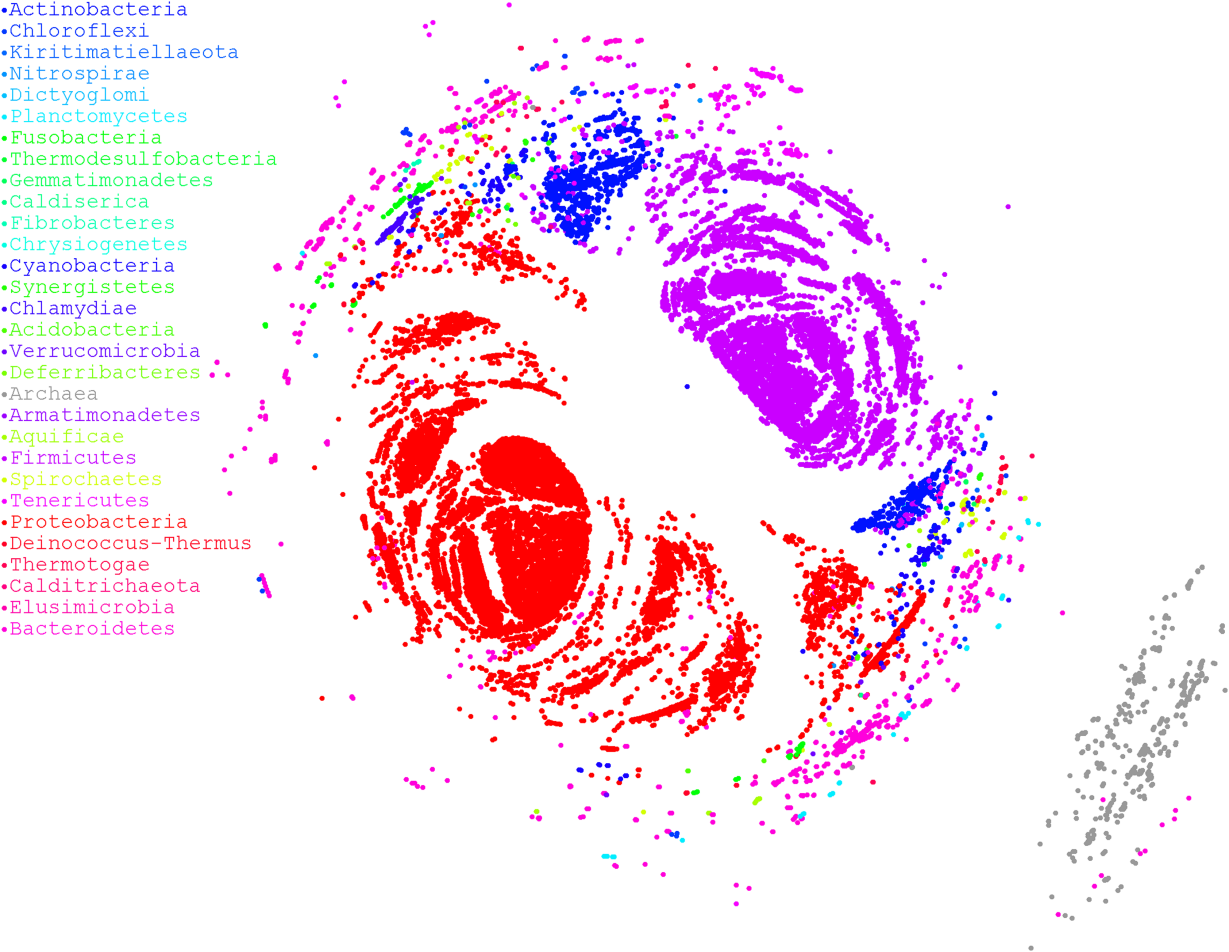
MDS ordination plot based on alignment distances among microorganisms. Different colors represent different phyla. A total of 38,360 sequences of 16S rRNA were downloaded from rrnDB, which contains 8,223 bacterial records representing 2,913 species and 262 archaeal records representing 201 species.

Finally, we used a large dataset to stress OutbreakFinder, which included 38,360 16S rRNA sequences downloaded from rrnDB (Stoddard et al., 2015). Although 38,360 sequences constitute a very large dataset, the results revealed that OutbreakFinder could still output results normally, as shown in Fig. 4. By contrast, if the traditional MDS method is used for dimension reduction analysis, more computational resources are required because the time complexity of traditional MDS is O(*n*^3^). In the case of NMDS, the time complexity is only O(cn^2^), where *c* is the number of iterations and usually does not exceed 1,000. Therefore, OutbreakFinder can calculate the MDS of 38,360 16S rRNA in 1 day using a single core and 30 G memory computing resources. In contrast, the manifold.mds cannot handle the same dataset, even if the computing resources are increased to 20 core and 120 G memory, the work cannot be completed. Manifold.mds is a method in the scikit-learn suite in Python. To confirm that the proposed method is robust enough, we performed 1,000 simulations to compare the performance of NMDS and manifold.mds. As shown in Fig. S2, in most of the simulated fn is smaller than fc, fn, and fc are the values of the error function of NMDS and manifold.mds, respectively, which means that the performance of NMDS is better than that of manifest.mds.

## Discussion

Application of phylogenetic information for epidemiological inference is increasingly popular in the next generation sequencing era. Data from several approaches such as PFGE, single nucleotide polymorphism (SNP), and MLST can be used to construct a phylogenetic tree. However, interpreting a phylogenetic tree without training in evolutionary biology is seldom easy. An epidemiologist’s primary goal is to determine whether bacterial isolates form a cluster that might indicate a probable outbreak. Therefore, development of a simple-to-use visualization tool that can help epidemiologists interpret relationships among compared isolates is crucial. In this paper, we present a visualization tool named OutbreakFinder that can show relationships among compared bacterial isolates on a two-dimensional MDS plot from distance matrix calculation or tree transformation (in Newick format). With the assistance of OutbreakFinder, epidemiologists can easily investigate probable outbreaks without experiencing the difficulty of “reading” phylogenetic trees.

## Conclusions

The analysis of differences among biological individuals based on inferred phylogenetic trees is one of the main methods. It is also often used to analysis outbreaks in epidemiology. Relative to the phylogenetic tree, the graph may be more suitable for the presentation of outbreak analysis. We recommend using the MDS method to present the results of the analysis, because MDS is more suitable for use in clustering problems. We use empirical data to verify the performance of OutbreakFinder, and the results show that MDS plot can clearly distinguish outbreaks. In this study, we also improved on the weaknesses of existing MDS tools. On the one hand, we have improved the effect of clustering, while the other is to reduce the need for computing resources. In addition, we implemented AP in OutbreakFinder to facilitate cluster analysis. No need to specify the number of clusters is the advantage of using an AP. The source code for OutbreakFinder is available on GitHub.

## Supplemental Information

10.7717/peerj.7600/supp-1 Supplemental Information 1 Comparison for MDS with the traditional MDS (A) and Newton’s method (B). The 100 points are generated by the function genSimulationData() as shown in Appendix B. (A) The traditional MDS is generated by Scikit-learn, which is a machine learning library for Python, as shown in Appendix C. It can be found that the points 9 and 3 are assigned to the wrong group. (B) The MDS by using Newton’s method (NMDS) can reduce the grouping errors.

10.7717/peerj.7600/supp-2 Supplemental Information 2 The distribution of fc-fn simulated 1000 times. The error functions fc and fn represent the MDS of the traditional and Newtonian methods, respectively.

10.7717/peerj.7600/supp-3 Supplemental Information 3 59 *Salmonella* Heidelberg isolates from three outbreaks with the same pulsed-field gel electrophoresis pattern.

10.7717/peerj.7600/supp-4 Supplemental Information 4 31 *Listeria monocytogenes* isolates from an outbreak and outgroup.

10.7717/peerj.7600/supp-5 Supplemental Information 5 22 *Campylobacter jejuni* isolates from an outbreak and outgroup.

10.7717/peerj.7600/supp-6 Supplemental Information 6 9 *Escherichia coli* isolates from an outbreak and outgroup.

10.7717/peerj.7600/supp-7 Supplemental Information 7 23 *Salmonella enterica* isolates from an outbreak and outgroup.

10.7717/peerj.7600/supp-8 Supplemental Information 8 The classical MDS method derivation.

10.7717/peerj.7600/supp-9 Supplemental Information 9 Java code for generate simulation data.

10.7717/peerj.7600/supp-10 Supplemental Information 10 Python code for MDS calculations.

## Additional Information and Declarations

### Data Availability

The following information was supplied regarding data availability: Data is available at GitHub: https://github.com/skypes/Newton-method-MDS.
