# Supplementary material for: OutbreakFinder: a visualization tool for rapid detection of bacterial strain clusters based on optimized multidimensional scaling
Source: PeerJ. 2019 Aug 28;7:e7600. doi: 10.7717/peerj.7600 (PMC6717506; doi:10.7717/peerj.7600)
Supplement: Supplemental Information 1 — The 100 points are generated by the function genSimulationData() as shown in Appendix B. (A) The traditional MDS is generated by Scikit-learn, which is a machine learning library for Python, as shown in Appendix C. It can be found that the points 9 and 3 are assigned to the wrong group. (B) The MDS by using Newton’s method (NMDS) can reduce the grouping errors. [file peerj-07-7600-s001.pdf]

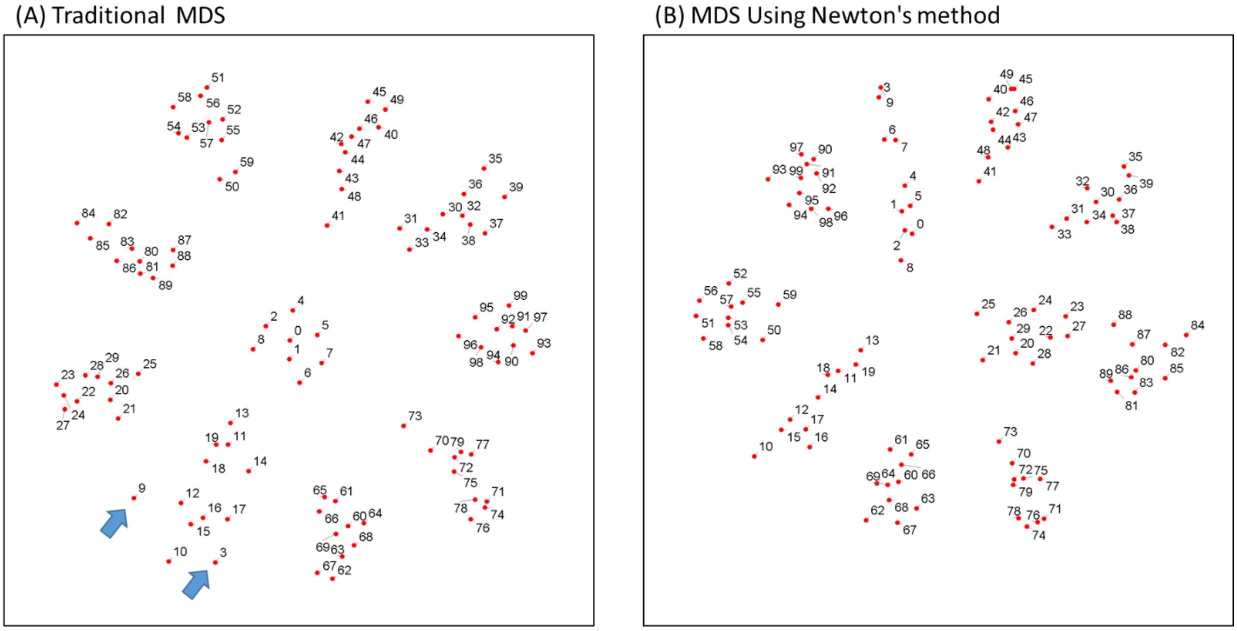

Figure S1. Comparison for MDS with the traditional MDS (A) and Newton's method (B). The 100 points are generated by the function genSimulationData() as shown in Supplementary Appendix B. (A) The traditional MDS is generated by Scikit-learn, which is a machine learning library for Python, as shown in Supplementary Appendix C. It can be found that the points 9 and 3 are assigned to the wrong group. (B) The MDS by using Newton’s method (NMDS) can reduce the grouping errors.
