# Supplementary material for: OutbreakFinder: a visualization tool for rapid detection of bacterial strain clusters based on optimized multidimensional scaling
Source: PeerJ. 2019 Aug 28;7:e7600. doi: 10.7717/peerj.7600 (PMC6717506; doi:10.7717/peerj.7600)
Supplement: Supplemental Information 2 — The error functions fc and fn represent the MDS of the traditional and Newtonian methods, respectively. [file peerj-07-7600-s002.pdf]

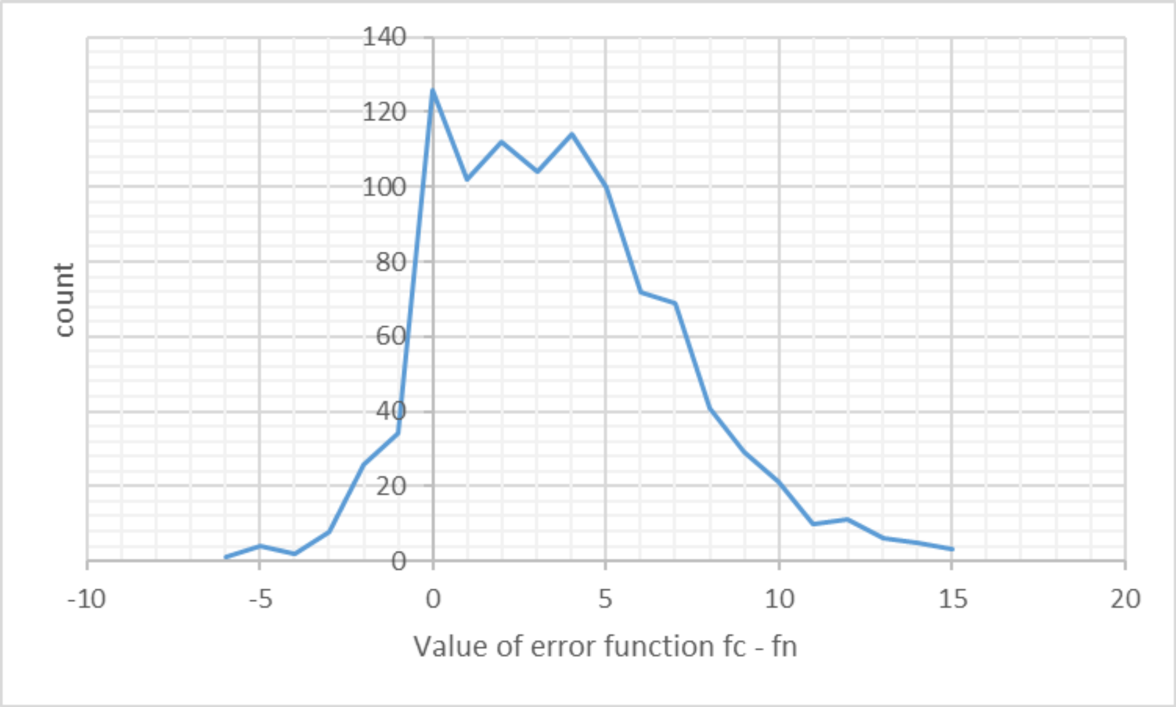

Figure S2. The distribution of fc-fn simulated 1000 times, where the error functions fc and fn represent the MDS of the traditional and Newtonian methods, respectively.
