# Supplementary material for: OutbreakFinder: a visualization tool for rapid detection of bacterial strain clusters based on optimized multidimensional scaling
Source: PeerJ. 2019 Aug 28;7:e7600. doi: 10.7717/peerj.7600 (PMC6717506; doi:10.7717/peerj.7600)
Supplement: Supplemental Information 3 [file peerj-07-7600-s003.docx]

**Table S1.** 59 *Salmonella* Heidelberg isolates from three outbreaks with the same pulsed-field gel electrophoresis pattern.

| **Label** | **Accession No.** | **Strain** | **Outbreak No.** |
| --- | --- | --- | --- |
| 1 | SRR3028736 | SH12-001 | Outbreak1 |
| 2 | SRR3028737 | SH12-002 | Outbreak1 |
| 3 | SRR3028738 | SH12-003 | Outbreak1 |
| 4 | SRR3028739 | SH12-004 | Outbreak1 |
| 5 | SRR3028740 | SH12-005 | Outbreak1 |
| 6 | SRR3028741 | SH12-006 | Outbreak1 |
| 7 | SRR3028742 | SH12-007 | Outbreak1 |
| 8 | SRR3028743 | SH12-008 | Outbreak1 |
| 9 | SRR3028744 | SH12-009 | Outbreak1 |
| 10 | SRR3028745 | SH12-010 | Outbreak1 |
| 11 | SRR3028746 | SH13-001 | Outbreak2 |
| 12 | SRR3028747 | SH13-002 | Outbreak2 |
| 13 | SRR3028748 | SH13-003 | Outbreak2 |
| 14 | SRR3028749 | SH13-004 | Outbreak2 |
| 15 | SRR3028750 | SH13-005 | Outbreak2 |
| 16 | SRR3028751 | SH13-006 | Outbreak2 |
| 17 | SRR3028752 | SH13-007 | Outbreak2 |
| 18 | SRR3028753 | SH13-008 | Outbreak2 |
| 19 | SRR3028754 | SH14-001 | Outbreak3 |
| 20 | SRR3028755 | SH14-002 | Outbreak3 |
| 21 | SRR3028756 | SH14-003 | Outbreak3 |
| 22 | SRR3028757 | SH14-004 | Outbreak3 |
| 23 | SRR3028758 | SH14-005 | Outbreak3 |
| 24 | SRR3028759 | SH14-006 | Outbreak3 |
| 25 | SRR3028760 | SH14-007 | Outbreak3 |
| 26 | SRR3028761 | SH14-008 | Outbreak3 |
| 27 | SRR3028762 | SH14-009 | Outbreak3 |
| 28 | SRR3028763 | SH14-010 | Outbreak3 |
| 29 | SRR3028764 | SH14-011 | Outbreak3 |
| 30 | SRR3028765 | SH14-012 | Outbreak3 |
| 31 | SRR3028766 | SH14-013 | Outbreak3 |
| 32 | SRR3028767 | SH14-014 | Outbreak3 |
| 33 | SRR3028768 | SH14-015 | Outbreak3 |
| 34 | SRR3028769 | SH14-016 | Outbreak3 |
| 35 | SRR3028770 | SH14-017 | Outbreak3 |
| 36 | SRR3028771 | SH14-018 | Outbreak3 |
| 37 | SRR3028772 | SH14-019 | Outbreak3 |
| 38 | SRR3028773 | SH14-020 | Outbreak3 |
| 39 | SRR3028774 | SH14-021 | Outbreak3 |
| 40 | SRR3028775 | SH14-022 | Outbreak3 |
| 41 | SRR3028776 | SH14-023 | Outbreak3 |
| 42 | SRR3028777 | SH14-024 | Outbreak3 |
| 43 | SRR3028778 | SH14-025 | Outbreak3 |
| 44 | SRR3028779 | SH14-026 | Outbreak3 |
| 45 | SRR3028780 | SH14-027 | Outbreak3 |
| 46 | SRR3028781 | SH14-028 | Outbreak3 |
| 47 | SRR3028782 | SH12-011 | - |
| 48 | SRR3028783 | SH10-001 | - |
| 49 | SRR3028784 | SH11-001 | - |
| 50 | SRR3028785 | SH10-002 | - |
| 51 | SRR3028786 | SH12-012 | - |
| 52 | SRR3028787 | SH12-014 | - |
| 53 | SRR3028788 | SH12-013 | - |
| 54 | SRR3028789 | SH10-014 | - |
| 55 | SRR3028790 | SH11-002 | - |
| 56 | SRR3028791 | SH10-015 | - |
| 57 | SRR3028792 | SH08-001 | - |
| 58 | SRR3028793 | SH09-29 | - |
| 59 | SRR3028794 | SH10-30 | - |
