# Supplementary material for: OutbreakFinder: a visualization tool for rapid detection of bacterial strain clusters based on optimized multidimensional scaling
Source: PeerJ. 2019 Aug 28;7:e7600. doi: 10.7717/peerj.7600 (PMC6717506; doi:10.7717/peerj.7600)
Supplement: Supplemental Information 4 [file peerj-07-7600-s004.docx]

**Table S2.** 31 *Listeria monocytogenes* isolates from an outbreak and outgroup.

| **Label** | **Accession No.** | **Strain** | **Outbreak No.** |
| --- | --- | --- | --- |
| 1 | SRR1206159 | CFSAN002349 | outgroup |
| 2 | SRR1393979 | PNUSAL000730 | outgroup |
| 3 | SRR1534987 | PNUSAL000870 | 1408MLGX6-3WGS |
| 4 | SRR1553739 | MOD1_LS982 | 1408MLGX6-3WGS |
| 5 | SRR1553756 | MOD1_LS996 | 1408MLGX6-3WGS |
| 6 | SRR1553773 | MOD1_LS1006 | 1408MLGX6-3WGS |
| 7 | SRR1553774 | MOD1_LS1011 | 1408MLGX6-3WGS |
| 8 | SRR1553788 | MOD1_LS997 | 1408MLGX6-3WGS |
| 9 | SRR1553791 | MOD1_LS994 | 1408MLGX6-3WGS |
| 10 | SRR1553792 | MOD1_LS1003 | 1408MLGX6-3WGS |
| 11 | SRR1553804 | MOD1_LS998 | 1408MLGX6-3WGS |
| 12 | SRR1553816 | MOD1_LS995 | 1408MLGX6-3WGS |
| 13 | SRR1553821 | MOD1_LS1010 | 1408MLGX6-3WGS |
| 14 | SRR1553826 | MOD1_LS1004 | 1408MLGX6-3WGS |
| 15 | SRR1553827 | MOD1_LS1009 | 1408MLGX6-3WGS |
| 16 | SRR1553851 | MOD1_LS1008 | 1408MLGX6-3WGS |
| 17 | SRR1553856 | MOD1_LS989 | 1408MLGX6-3WGS |
| 18 | SRR1553867 | MOD1_LS1005 | 1408MLGX6-3WGS |
| 19 | SRR1553882 | MOD1_LS985 | 1408MLGX6-3WGS |
| 20 | SRR1553907 | MOD1_LS1000 | 1408MLGX6-3WGS |
| 21 | SRR1556288 | CFSAN023463 | 1408MLGX6-3WGS |
| 22 | SRR1556289 | CFSAN023466 | 1408MLGX6-3WGS |
| 23 | SRR1556290 | CFSAN023468 | 1408MLGX6-3WGS |
| 24 | SRR1556291 | CFSAN023469 | 1408MLGX6-3WGS |
| 25 | SRR1556293 | CFSAN023465 | 1408MLGX6-3WGS |
| 26 | SRR1556294 | CFSAN023464 | 1408MLGX6-3WGS |
| 27 | SRR1556295 | CFSAN023471 | 1408MLGX6-3WGS |
| 28 | SRR1556296 | CFSAN023467 | 1408MLGX6-3WGS |
| 29 | SRR1556297 | CFSAN023470 | 1408MLGX6-3WGS |
| 30 | SRR1562157 | PNUSAL000957 | outgroup |
| 31 | SRR1597487 | PNUSAL001024 | 1408MLGX6-3WGS |
