# Supplementary material for: OutbreakFinder: a visualization tool for rapid detection of bacterial strain clusters based on optimized multidimensional scaling
Source: PeerJ. 2019 Aug 28;7:e7600. doi: 10.7717/peerj.7600 (PMC6717506; doi:10.7717/peerj.7600)
Supplement: Supplemental Information 5 [file peerj-07-7600-s005.docx]

**Table S3.** 22 *Campylobacter jejuni* isolates from an outbreak and outgroup.

| **Label** | **Accession No.** | **Strain** | **Outbreak No.** |
| --- | --- | --- | --- |
| 1 | SRR1993270 | 2014D-0067 | outgroup |
| 2 | SRR1993271 | 2014D-0068 | outgroup |
| 3 | SRR1993272 | 2014D-0070 | outgroup |
| 4 | SRR1999649 | D5663 | outgroup |
| 5 | SRR1999661 | 2014D-0189 | outgroup |
| 6 | SRR2984947 | PNUSA000196 | outgroup |
| 7 | SRR2985018 | PNUSA000195 | outgroup |
| 8 | SRR2985019 | PNUSA000194 | outgroup |
| 9 | SRR3214715 | D7331 | 0810PADBR-1 |
| 10 | SRR3215024 | D7330 | 0810PADBR-1 |
| 11 | SRR3215107 | D7329 | 0810PADBR-1 |
| 12 | SRR3215108 | D7328 | 0810PADBR-1 |
| 13 | SRR3215123 | D7324 | 0810PADBR-1 |
| 14 | SRR3215124 | D7323 | 0810PADBR-1 |
| 15 | SRR3215135 | D7321 | 0810PADBR-1 |
| 16 | SRR3215209 | D7322 | 0810PADBR-1 |
| 17 | SRR3215210 | D7319 | 0810PADBR-1 |
| 18 | SRR3215211 | D7327 | 0810PADBR-1 |
| 19 | SRR3216118 | D7333 | 0810PADBR-1 |
| 20 | SRR3216133 | D7316 | 0810PADBR-1 |
| 21 | SRR3216186 | D7320 | 0810PADBR-1 |
| 22 | SRR3216366 | D7334 | 0810PADBR-1 |
