# Supplementary material for: OutbreakFinder: a visualization tool for rapid detection of bacterial strain clusters based on optimized multidimensional scaling
Source: PeerJ. 2019 Aug 28;7:e7600. doi: 10.7717/peerj.7600 (PMC6717506; doi:10.7717/peerj.7600)
Supplement: Supplemental Information 6 [file peerj-07-7600-s006.docx]

**Table S4.** 9 *Escherichia coli* isolates from an outbreak and outgroup.

| **Label** | **Accession No.** | **Strain** | **Outbreak No.** |
| --- | --- | --- | --- |
| 1 | SRR1609861 | 1405WAEXK-1 | 1405WAEXK-1 |
| 2 | SRR1609862 | 1405WAEXK-1 | 1405WAEXK-1 |
| 3 | SRR1609871 | 1405WAEXK-1 | 1405WAEXK-1 |
| 4 | SRR1610028 | 1405WAEXK-1 | outgroup |
| 5 | SRR1610029 | 1405WAEXK-1 | outgroup |
| 6 | SRR1610031 | 1405WAEXK-1 | outgroup |
| 7 | SRR1610032 | 1405WAEXK-1 | outgroup |
| 8 | SRR1610033 | 1405WAEXK-1 | outgroup |
| 9 | SRR1610034 | 1405WAEXK-1 | outgroup |
