# Supplementary material for: OutbreakFinder: a visualization tool for rapid detection of bacterial strain clusters based on optimized multidimensional scaling
Source: PeerJ. 2019 Aug 28;7:e7600. doi: 10.7717/peerj.7600 (PMC6717506; doi:10.7717/peerj.7600)
Supplement: Supplemental Information 7 [file peerj-07-7600-s007.docx]

**Table S5.** 23 *Salmonella enterica* isolates from an outbreak and outgroup.

| **Label** | **Accession No.** | **Strain** | **Outbreak No.** |
| --- | --- | --- | --- |
| 1 | SRR1258439 | CFSAN001112 | 1203NYJAP-1 |
| 2 | SRR1258440 | CFSAN001140 | 1203NYJAP-1 |
| 3 | SRR1258442 | CFSAN001115 | 1203NYJAP-1 |
| 4 | SRR1258443 | CFSAN001118 | 1203NYJAP-1 |
| 5 | SRR498276 | CFSAN000189 | outgroup |
| 6 | SRR498369 | CFSAN000191 | outgroup |
| 7 | SRR498373 | CFSAN000211 | outgroup |
| 8 | SRR498397 | CFSAN000661 | 1203NYJAP-1 |
| 9 | SRR498399 | CFSAN000669 | 1203NYJAP-1 |
| 10 | SRR498402 | CFSAN000700 | 1203NYJAP-1 |
| 11 | SRR498403 | CFSAN000752 | 1203NYJAP-1 |
| 12 | SRR498404 | CFSAN000753 | 1203NYJAP-1 |
| 13 | SRR498422 | CFSAN000951 | 1203NYJAP-1 |
| 14 | SRR498423 | CFSAN000952 | 1203NYJAP-1 |
| 15 | SRR498425 | CFSAN000954 | 1203NYJAP-1 |
| 16 | SRR498431 | CFSAN000958 | 1203NYJAP-1 |
| 17 | SRR498433 | CFSAN000960 | 1203NYJAP-1 |
| 18 | SRR498434 | CFSAN000961 | 1203NYJAP-1 |
| 19 | SRR498436 | CFSAN000963 | 1203NYJAP-1 |
| 20 | SRR498442 | CFSAN000968 | 1203NYJAP-1 |
| 21 | SRR498444 | CFSAN000970 | 1203NYJAP-1 |
| 22 | SRR500493 | CFSAN000228 | outgroup |
| 23 | SRR500494 | CFSAN000212 | outgroup |
