# Supplementary material for: OutbreakFinder: a visualization tool for rapid detection of bacterial strain clusters based on optimized multidimensional scaling
Source: PeerJ. 2019 Aug 28;7:e7600. doi: 10.7717/peerj.7600 (PMC6717506; doi:10.7717/peerj.7600)
Supplement: Supplemental Information 8 [file peerj-07-7600-s008.pdf]

Appendix A. The MDS method derivation.

Given *n* points $\{X_1, X_2 \dots X_n\}$ in w-dimensional space, where

$$X_i = \begin{bmatrix} x_{i1} \\ x_{i2} \\ \vdots \\ x_{iw} \end{bmatrix}$$

And the Euclidean distance between node X_i and X_j should be

$$d_{ij}^2 = \sum_{k=1}^{w} (X_{ik} - X_{jk})^2 = \|X_i - X_j\|^2 = (X_i - X_j)^T (X_i - X_j)$$

Let M be an inner product matrix and $m_{ij} = X_i^T X_j$, where m_{ij} is an inner product of X_i and X_j. M is a symmetric matrix, therefor $X_i^T X_j = X_j^T X_i$. The matric M is shown below:

$$\mathrm{M} = \begin{bmatrix} m_{11} & \cdots & m_{1n} \\ \vdots & \ddots & \vdots \\ m_{n1} & \cdots & m_{nn} \end{bmatrix}$$

Assuming centered configuration to excluding the effect of coordinate translation:

$$\sum_{k=1}^{n} X_k = 0$$

Expanding the formula for Euclidean distance:

$$d_{ij}^2 = X_i^T X_i + X_j^T X_j - 2X_i^T X_j$$

$$\frac{1}{n}\sum_{i=1}^{n} d_{ij}^2 = \frac{1}{n}\sum_{i=1}^{n} \left(X_i^T X_i + X_j^T X_j - 2X_i^T X_j\right)$$

$$= \frac{1}{n}\left(\sum_{i=1}^{n} X_i^T X_i + nX_j^T X_j - 2\left(\sum_{i=1}^{n} X_i\right)^T X_j\right)$$

Then, the formula (1) and (2) are derived:

$$\frac{1}{n}\sum_{i=1}^{n} d_{ij}^2 = \frac{1}{n}\sum_{i=1}^{n} X_i^T X_i + X_j^T X_j \qquad (1)$$

$$\frac{1}{n}\sum_{j=1}^{n} d_{ij}^2 = X_i^T X_i + \frac{1}{n}\sum_{j=1}^{n} X_j^T X_j \qquad (2)$$

Expanding the formula of Euclidean distance for all d_{ij}:

$$\frac{1}{n^2}\sum_{i=1}^{n}\sum_{j=1}^{n} d_{ij}^2 = \frac{1}{n^2}\sum_{i=1}^{n}\sum_{j=1}^{n}(X_i^T X_i + X_j^T X_j - 2X_i^T X_j)$$

$$= \frac{1}{n^2}\left(n\sum_{i=1}^{n} X_i^T X_i + n\sum_{j=1}^{n} X_j^T X_j - 2\sum_{j=1}^{n}\left(\sum_{i=1}^{n} X_i\right)^T X_j\right)$$

Then, the formula (3) is derived:

$$\frac{1}{n^2}\sum_{i=1}^{n}\sum_{j=1}^{n} d_{ij}^2 = \frac{2}{n}\sum_{i=1}^{n} X_i^T X_i \qquad (3)$$

Solve the following equations by using formula (1)+(2)-(3)

$$X_i^T X_i + X_j^T X_j = \frac{1}{n}\sum_{i=1}^{n} d_{ij}^2 + \frac{1}{n}\sum_{j=1}^{n} d_{ij}^2 - \frac{1}{n^2}\sum_{i=1}^{n}\sum_{j=1}^{n} d_{ij}^2 \qquad (4)$$

Bring d_{ij}^2 into formula (4):

$$m_{ij} = X_i^T X_j$$

$$= -\frac{1}{2}(d_{ij}^2 - X_i^T X_i - X_j^T X_j)$$

$$= -\frac{1}{2}\left(d_{ij}^2 - \frac{1}{n}\sum_{i=1}^{n} d_{ij}^2 - \frac{1}{n}\sum_{j=1}^{n} d_{ij}^2 + \frac{1}{n^2}\sum_{i=1}^{n}\sum_{j=1}^{n} d_{ij}^2\right)$$

Above formula can be translate into simple form, let $b_{ij} = -\frac{1}{2}d_{ij}^2$, and then we can denote $b_{i\_}$, $b_{\_j}$ and $b_{\_}$ as following:

$$b_{i\_} = \frac{1}{n}\sum_{j=1}^{n} b_{ij}$$

$$b_{\_j} = \frac{1}{n}\sum_{i=1}^{n} b_{ij}$$

$$b\_ = \frac{1}{n^2}\sum_{j=1}^{n}\sum_{i=0}^{n} b_{ij}$$

Therefore, we got following formulas:

$$m_{ij} = b_{ij} - b_{i\_} - b_{\_j} + b\_$$

$$M = \begin{bmatrix} b_{11} - b_{1\_} - b_{\_1} + b\_ & \cdots & b_{1n} - b_{1\_} - b_{\_n} + b\_ \\ \vdots & \ddots & \vdots \\ b_{n1} - b_{n\_} - b_{\_1} + b\_ & \cdots & b_{nn} - b_{n\_} - b_{\_n} + b\_ \end{bmatrix}$$

M is a symmetric matrix, which can be translate into following form:

$$M = UAU$$

Substituting $U = I - \frac{1}{n}E$ directly into $M = UAU$:

$$M = \left(I - \frac{1}{n}E\right)A\left(I - \frac{1}{n}E\right) = A - \frac{1}{n}EA - \frac{1}{n}AE - \frac{1}{n^2}EAE$$

In metric MDS problem, the matrix M can be reconstructed based on the following spectral decomposition and Cholesky decomposition:

$$M = VCV^T = V\sqrt{C}\sqrt{C}V^T = \left(\sqrt{C}V^T\right)^T\left(\sqrt{C}V^T\right)$$

Where V is eigenvectors and C is diagonal matrix of eigenvalue. M is an inner product matrix, which can be represented as following form:

$$M = \begin{bmatrix} x_1^T \\ x_2^T \\ \vdots \\ x_n^T \end{bmatrix} [x_1 \quad x_2 \quad \cdots \quad x_n] = XX^T$$

Therefore, we got new coordinates of all points X by following equation:

$$X = V\sqrt{C}$$
