# Supplementary material for: OutbreakFinder: a visualization tool for rapid detection of bacterial strain clusters based on optimized multidimensional scaling
Source: PeerJ. 2019 Aug 28;7:e7600. doi: 10.7717/peerj.7600 (PMC6717506; doi:10.7717/peerj.7600)
Supplement: Supplemental Information 9 [file peerj-07-7600-s009.pdf]

Appendix B. Java code for generate simulation data.

```
public double[][] genSimulationData() throws Exception {
    int size = 100;
    int md = 10;
    double[][] simCoods = new double[size][md];
    for(int i=0; i<size; i++){
        for(int j=0; j<md; j++){
            double cx = Math.random()*3;
            simCoods[i][j]=cx;
        }
        int d = i/10;
        simCoods[i][d]+=14;
    }
    double[][] obdist = new double[size][size];
    for(int i=0; i<size; i++){
        for(int j=i; j<size; j++){
            double sum = 0;
            for(int d=0; d<md; d++){
                sum += Math.pow(simCoods[i][d]-simCoods[j][d], 2);
            }
            double dst = Math.pow(sum, 0.5);
            obdist[i][j] = dst;
            obdist[j][i] = dst;
        }
    }
    return obdist;
}
```
