# Supplementary material for: OutbreakFinder: a visualization tool for rapid detection of bacterial strain clusters based on optimized multidimensional scaling
Source: PeerJ. 2019 Aug 28;7:e7600. doi: 10.7717/peerj.7600 (PMC6717506; doi:10.7717/peerj.7600)
Supplement: Supplemental Information 10 [file peerj-07-7600-s010.pdf]

Appendix C. Python code for MDS calculations.

```
import os
import numpy as np

from matplotlib import pyplot as plt
from matplotlib.collections import LineCollection

from sklearn import manifold
from sklearn.metrics import euclidean_distances
from sklearn.decomposition import PCA


print(__doc__)

rootdir = 'E:\\home\\tmp2\\MDS\\1000test\\data'
dist = 'E:\\home\\tmp2\\MDS\\1000test\\pythonResult'

def runMDS( parameters, distFile ):
    seed = np.random.RandomState(seed=3)
    f = open(parameters, 'r')
    line = f.readline()
    sa = line.strip(' \n\r').split('\t')
    n_samples = len(sa)
    #print(n_samples)

    arr = [[None]]*n_samples
    for x in range(n_samples):
        arr[x] = [None]*n_samples
    for x in range(n_samples):
        arr[0][x] = np.float(sa[x])

    ptr = 1;
    for line in f:
        sa = line.strip(' \n\r').split('\t')
        for x in range(n_samples):
            arr[ptr][x] = np.float(sa[x])
        ptr+=1
```

```
    f.close()

    similarities = np.array(arr)

    #print(similarities)

    mds = manifold.MDS(n_components=2, max_iter=3000, eps=1e-9, random_state=seed,
                       dissimilarity="precomputed", n_jobs=1)
    pos = mds.fit(similarities).embedding_

    f2 = open(distFile, 'w')
    for ps in pos:
        #print(ps[0]+'\t'+ps[1]+'\n')
        f2.write(str(ps[0])+'\t'+str(ps[1])+'\n')
    f2.close()

    return parameters


for filename in os.listdir(rootdir):
    print(runMDS(rootdir+"\\"+filename, dist+"\\"+filename))
```
